# Gelatin-modified 3D printed PGS elastic hierarchical porous scaffold for cartilage regeneration

**DOI:** 10.1063/5.0152151

**Published:** 2023-08-04

**Authors:** Sinan Wang, Hongying Chen, Jinyi Huang, Sisi Shen, Zhengya Tang, Xiaoyan Tan, Dong Lei, Guangdong Zhou

**Affiliations:** 1 Research Institute of Plastic Surgery, Wei Fang Medical College, Weifang, China; 2 Department of Plastic and Reconstructive Surgery, Department of Cardiology, Shanghai Key Lab of Tissue Engineering, Shanghai 9th People's Hospital, Shanghai Jiao Tong University School of Medicine, Shanghai 200011, People's Republic of China; 3 Department of Human Anatomy, Xinxiang Medical University, Xinxiang, China

## Abstract

Regenerative cartilage replacements are increasingly required in clinical settings for various defect repairs, including bronchial cartilage deficiency, articular cartilage injury, and microtia reconstruction. Poly (glycerol sebacate) (PGS) is a widely used bioelastomer that has been developed for various regenerative medicine applications because of its excellent elasticity, biodegradability, and biocompatibility. However, because of inadequate active groups, strong hydrophobicity, and limited ink extrusion accuracy, 3D printed PGS scaffolds may cause insufficient bioactivity, inefficient cell inoculation, and inconsistent cellular composition, which seriously hinders its further cartilage regenerative application. Here, we combined 3D printed PGS frameworks with an encapsulated gelatin hydrogel to fabricate a PGS@Gel composite scaffold. PGS@Gel scaffolds have a controllable porous microstructure, with suitable pore sizes and enhanced hydrophilia, which could significantly promote the cells' penetration and adhesion for efficient chondrocyte inoculation. Furthermore, the outstanding elasticity and fatigue durability of the PGS framework enabled the regenerated cartilage built by the PGS@Gel scaffolds to resist the dynamic *in vivo* environment and maintain its original morphology. Importantly, PGS@Gel scaffolds increased the rate of cartilage regeneration concurrent with scaffold degradation. The scaffold was gradually degraded and integrated to form uniform, dense, and mature regenerated cartilage tissue with little scaffold residue.

## I. INTRODUCTION

Cartilage repair and regeneration remain a substantial challenge in clinical applications that usually requires surgical intervention because of the limited self-repair ability of cartilage tissue.^1^ Advanced surgical approaches for treating cartilage lesions, such as autologous cartilage or chondrocyte transplantation, help to improve function; however, they are limited by the availability of autograft cartilage or chondrocytes, and chondrocyte dedifferentiation during the *in vitro* culture process.^2,3^ Given the drawbacks with current interventions, scaffold-based tissue engineering has emerged and developed rapidly to address these challenges, aiming to regenerate tissues with the composition, structure, and functional characteristics close to that of the natural cartilage.^4–7^ Due to the precise control of microstructure and personalized customization, three-dimensional (3D) printing technology has been widely applied to produce tissue- engineering scaffolds for cartilage regeneration.^8,9^

Cartilage is predominantly composed of abundant extracellular matrix (ECM) wherein proteoglycans (PGs) and glycosaminoglycans (GAGs) are filled in a complex molecular network primarily consisted of interwoven type II collagen. Thereby, cartilage possesses both viscoelasticity and poroelasticity provided by its unique physiological structure, which is conducive for sustaining dynamic compressive stress cycles while maintaining its structure and mechanical stability in a complex mechanical microenvironment.^10,11^ Hence, biomaterial scaffolds should be designed to closely mimic the mechanical and biological properties of natural cartilage.^12–14^ Known for good deformability, biocompatibility, and biodegradation, bioelastomers are not only able to simulate the mechanical characteristics of human soft tissue but also maintain their structural and mechanical stability in a dynamic mechanical environment *in vivo*, presenting great application prospects for use in tissue engineering.^15,16^ Poly (glycerol sebacate) (PGS) is a bioelastomer that has been widely used in tissue regeneration applications.^17–19^ Recently, a type of PGS-based photocurable biological glue was developed for skin or vascular tissue repair and approved by the FDA in 2020, highlighting PGS as a promising prospect for clinical transformation in the future.^20^ Furthermore, based on our previous work, PGS can be successfully 3D printed with excellent elasticity and fatigue durability, as well as hierarchical porous structures, and employed for cardiac repair and trachea regeneration.^21–24^

As a key component of tissue engineering, scaffolds incorporating cells and bioactive molecules are usually designed to mimic native tissue microenvironments.^25,26^ 3D porous scaffolds play a vital role in tissue engineering. They need to provide adequate porosity through interconnected pores of a suitable size (similar to that of the native tissue), which can accommodate cell adhesion, proliferation, and migration, in addition to allowing efficient nutrient exchange.^27–29^ Generally, a small pore size is not conducive to the uniform distribution of cells and the inward growth of tissue. On the contrary, a large pore size can result in a large number of cell loss. Numerous studies have shown that the optimal size range for the interconnected micropores of cartilage tissue engineering scaffolds is 150–250 *μ*m.^30–32^ However, it is difficult to obtain precise microporous structure mainly restricted by the accuracy of extrusion-based 3D printing and the Barus effect of inks. Consequently, the pore size of 3D printed PGS scaffolds is usually ≥300 *μ*m, which leads to inefficient cell seeding. Additionally, the hydrophobicity of PGS has an adverse effect on cell adhesion and migration, resulting in agglomerated growth during *in vitro* culture. Therefore, it is crucial for 3D printed PGS scaffolds to be constructed with the appropriate biomimetic porous structure and hydrophilicity to effectively guide chondrocyte fate and induce high quality cartilage regeneration.

In general, salt leaching, freeze-drying, electrospinning, and 3D printing are the common approaches for fabricating 3D porous scaffolds. In our previous study, we successfully regenerated tracheal cartilage based on double-layered PGS-gelatin tubular scaffolds.^24^ The incorporation of electrospun gelatin fibers enhanced the hydrophilicity of the 3D printed PGS scaffolds and improved the chondrocyte, loading capacity. However, because of the heterogeneous material composition and nanoscale pore size, the chondrocytes mostly grew in the exterior electrospun fibrous layer instead of the interior 3D printed scaffold. Regenerated tracheal cartilage had inferior tissue integration with 3D printed PGS framework and poor shape maintenance. At present, filling hydrogels into the 3D printed framework is a popular strategy to obtain a composite scaffold with adjustable structural, mechanical, and biological features.^33–35^ More importantly, 3D printed PGS frameworks with hydrogels using a freeze-drying method to produce scaffolds for use in cartilage regeneration has not yet been reported.

**FIG. 1.**
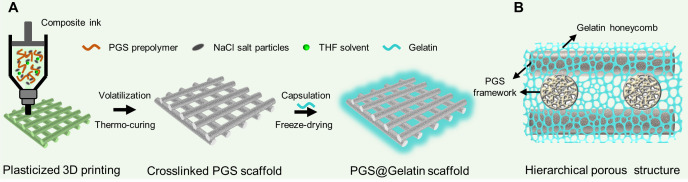
Schematic of the PGS@Gel hierarchical microporous scaffolds. (a) Illustration of the fabrication process. Composite inks of synthetic PGS prepolymer with salt particles and THF solvent were 3D printed and thermos-crosslinked to build a PGS framework. Gelatin was encapsulated and lyophilized in the PGS framework to prepare a PGS@Gel scaffold. (b) Gelatin porous networks filled and fixed into the interstitial space between 3D printed filaments to build a composite multi-level porous structure.

In this study, we combined 3D printed PGS scaffolds with gelatin (Gel) using hydrogel encapsulation and freeze-drying to fabricate a PGS@Gel composite scaffold [Figs. 1(a) and 1(b)]. This complementary design endowed PGS@Gel scaffolds with a well-organized hierarchical porous structure, enhanced hydrophilicity, excellent elasticity, and fatigue durability. Importantly, the PGS@Gel scaffolds promoted the adhesion, migration, and proliferation of chondrocytes, as well as the synthesis of ECM, *in vitro*. Furthermore, the regenerated cartilage built by the PGS@Gel scaffold matured gradually and was homogeneous with elasticity close to that of the native cartilage. This can be attributed to the coordinating rates of cartilage integration and PGS degradation that was observed *in vivo*. Our design provides a feasible strategy for cartilage regeneration, focusing on a biomimetic approach to elasticity and structure, and could be potentially applied to the repair and reconstruction of other complex tissues and organs in the future.

## II. RESULTS AND DISCUSSION

### A. Fabrication and characterization of PGS@Gel scaffolds

To ensure uniform mixing of the PGS with the salt particles and to improve the 3D printability of the composite ink, a solvent (THF) was added to prepare the paste ink. Compared with the heating and melting mixing method, the plasticization of a solvent can significantly improve the rheology, extrudability, and stability of the ink.^36^ It seems that the pore size of the PGS scaffold can be adjusted to 150–250 *μ*m (suitable for cell seeding) by adjusting the 3D print filling path parameters. However, this will decrease porosity of scaffold while greatly increase the printing accuracy requirements and difficulties. Importantly, it is easy to form an air film or air bubbles on the surface and inside of the scaffold under the synergetic effects of PGS hydrophobicity and rough surface microstructure, the surface tension will greatly limit the internal infiltration and inoculation efficiency of the chondrocytes. Compared with a composite scaffold fabricated using electrospinning,^24^ the PGS@Gel scaffold possessed a more homogeneous porous structure rather than a multi-layered structure, which would be beneficial for the uniform distribution of chondrocytes. Furthermore, this strategy is highly versatile and could be applied to other natural materials and drugs to fabricate functional 3D printed PGS scaffolds [Figs. 1(a) and 1(b)].

**FIG. 2.**
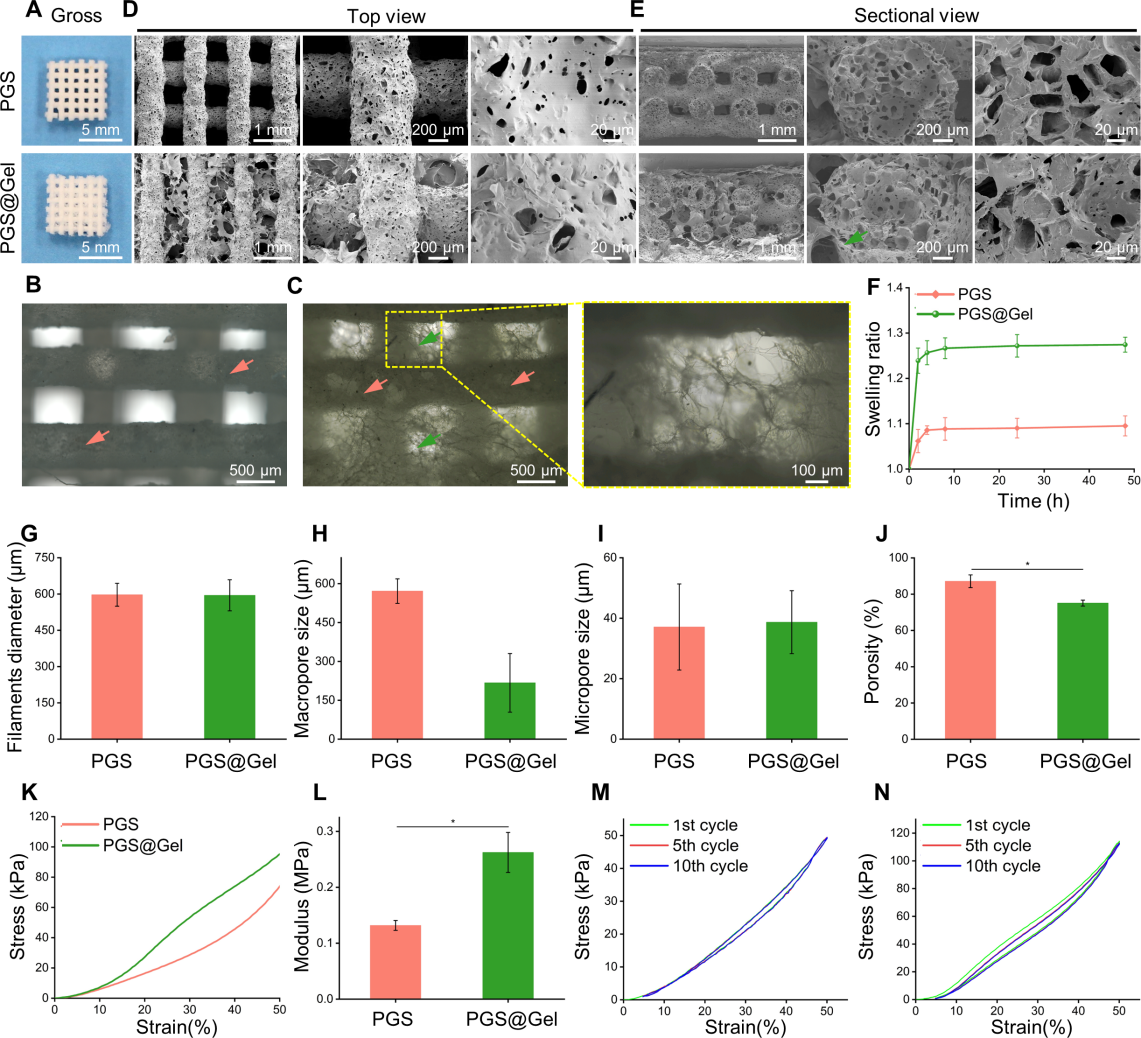
Morphologies and mechanical properties of the PGS@Gel scaffolds. (a) Optical images of a 3D printed PGS scaffold and PGS@Gel scaffold. Scale bars: 5 mm. (b) and (c) Optical microscope images of the PGS (b) and PGS@Gel scaffolds (c). Red and green arrows represent PGS framework and gelatin fibrous network, respectively. Scale bars: 500 and 100 *μ*m. (d) and (e) SEM images of PGS and PGS@Gel scaffolds at different magnifications in top view (d) and section view (e). Scale bars: 1 mm, 200, and 20 *μ*m. (f) Swelling ratio of PGS and PGS@Gel scaffolds. (g) Diameters of 3D printed PGS filaments. (h) Macropore size of the PGS framework and gelatin fibrous network. (i) Micropore size inside the filaments. (j) Porosity of PGS and PGS@Gel scaffolds. (k) Typical compressive stress–strain curves of scaffolds in a dry state. (l) Comparison of elastic modulus from compression tests. (m) and (n) Cyclic compression tests of PGS (m) and PGS@Gel (n) scaffolds for ten cycles with maximum strain of 50%. ^*^P < 0.05.

After encapsulation, freeze-drying, and cross-linking, PGS@Gel scaffolds still maintained the initial morphological features of the PGS scaffold, with gelatin evenly filled into the interstitial areas [Figs. 2(a)–2(c)]. SEM images demonstrated that gelatin was present throughout and integrated into the PGS framework with well-organized hierarchical structures [Figs. 2(d) and 2(e)]. The swelling ratio of PGS and PGS@Gel scaffolds were 1.08 ± 0.025 and 1.27 ± 0.023 [Fig. 2(f)]. PGS basically did not swell in the PBS due to its hydrophobicity. Compared with PGS scaffold, PGS@Gel had a higher swelling ratio, attributed to the significant swelling properties of hydrophilic gelatin in water. A multi-layered PGS framework was formed with vertically stacked filaments. The 3D printed filament diameters of the PGS and PGS@Gel scaffolds were 597.15 ± 47.31 and 594.70 ± 64.21 *μ*m, respectively [Fig. 2(g)]. Compared with the macropores of the PGS framework (pore size = 571.32 ± 47.41 *μ*m), gelatin fibrous networks were uniformly distributed between PGS filaments to provide smaller macropores (pore size =217.35 ± 113.12 *μ*m) [Fig. 2(h)]. During the cell inoculation and *in vitro* culture, the cells adhered to and grew in the macropores of the scaffold. The macropore size of the PGS@Gel scaffold was more adapted to the appropriate pore size (about 150–250 *μ*m) for cell growth, but the PGS scaffold macropore size was much larger. Furthermore, there were plenty of cubic micropores obtained by salt leaching, existing on the surface and inside of 3D printed filaments. The micropore sizes of the PGS and PGS@Gel scaffolds were 37.09 ± 14.24 and 38.69 ± 10.42 *μ*m, respectively, and determined by the salt particulate size, which could be adjusted by the grinding and sieving process during preparation [Fig. 2(i)]. These micropores provided channels for chondrocytes to migrate and grow in PGS filaments *in vivo*. The porosity of the PGS@Gel scaffold (75.08 ± 1.64%) was decreased as gelatin fibrous networks added in PGS scaffold [87.13 ± 3.51%, Fig. 2(j)]. In addition, this fibrous gelatin could modify the hydrophilicity of the PGS scaffold surface to mimic that of native ECM, which could help enable the PGS@Gel hybrid scaffold to promote various cell behaviors, including penetration, adhesion, proliferation, and matrix secretion.

Natural cartilage has excellent elasticity because of the large amount of elastic polysaccharide and collagen ECM. The addition of gelatin significantly improved the stiffness of the PGS scaffolds in a dry state because of the hydrogen bond interactions of amino and hydroxyl groups on the molecular chain of the gelatin [Fig. 2(k)]. The uniaxial compression tests established that the PGS and PGS@Gel scaffolds had an elastic modulus of 0.13 ± 0.01 and 0.26 ± 0.04 MPa, respectively [Fig. 2(l)]. Furthermore, cyclic compressive tests determined that the PGS and PGS@Gel scaffolds had excellent elasticity and anti-fatigue properties with limited hysteresis under dynamic pressure [Figs. 2(m) and 2(n)]. The elasticity and fatigue durability of the scaffold enabled the regenerated cartilage tissue to withstand multiple dynamic deformations and maintain its original morphology after implantation.

### B. *In vitro* cartilage regeneration using PGS@Gel scaffolds

**FIG. 3.**
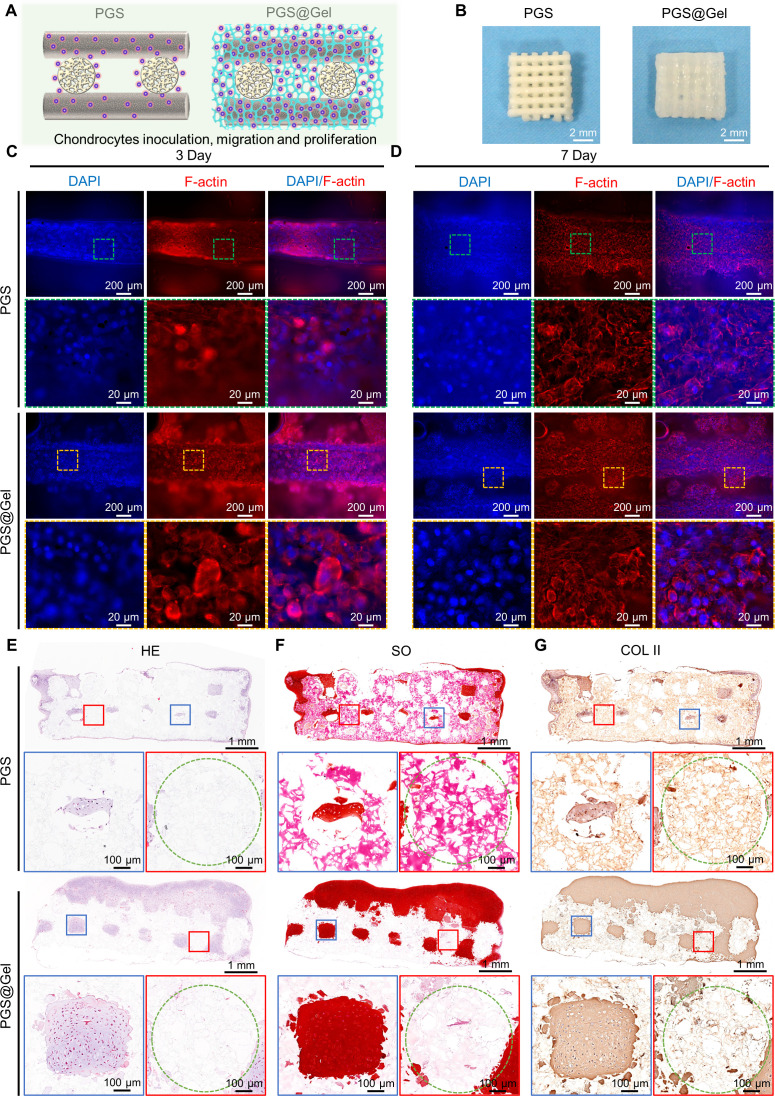
Biocompatibility and cartilage regeneration *in vitro* culture. (a) Schematic of chondrocyte seeding of the PGS and PGS@Gel scaffolds. (b) Optical images of engineered cartilages after 4 weeks *in vitro* culture. Scale bar: 2 mm. (c) and (d) Immunofluorescence staining images of cell-scaffolds at 3 days (c) and 7 days (d) *in vitro* culture. Red: F-actin, blue: DAPI; scale bar: 200, 20 *μ*m. (e)–(g) Hematoxylin and eosin (HE), Safranin-O (SO), and immunohistochemical collagen II (COL II) staining of regenerated cartilage in sectional view after 4 weeks *in vitro*. Blue rectangle: higher magnification of cartilage tissue in an interstitial area. Red rectangle: higher magnification of cartilage tissue in a filament area. Green circle: outline of scaffold filaments. Scale bar: 1 mm and 100 *μ*m.

To evaluate the ability of the PGS@Gel composite scaffolds to support cell growth, chondrocytes were dropwise seeded onto PGS and PGS@Gel scaffolds. Immunofluorescence staining showed that PGS@Gel scaffold could significantly promoted the adhesion and proliferation of chondrocytes than that of the PGS scaffold [Figs. 3(c) and 3(d)]. Chondrocytes only adhered and grew on the 3D printed PGS filaments in PGS group, but more chondrocytes grew on 3D printed PGS framework and gelatin fibrous network in PGS@Gel group. After 4 weeks of *in vitro* culture, both the PGS and PGS@Gel groups successfully generated cartilage-like tissues and maintained their original shape. Notably, the cartilage-scaffold construct of the PGS@Gel group was evenly filled with regenerated tissues compared with the PGS group, which still had many holes [Fig. 3(b)]. The sectional view of the histological analysis further confirmed that samples of both groups had started to form the typical cartilage-specific lacunae structure with ECM deposition, as revealed by HE, SO, and COL II staining [Figs. 3(e)–3(g)]. In addition, the outline of PGS filaments was still distinct without significant degradation and cell invasion. Importantly, the PGS@Gel group exhibited a more extensive and homogeneous distribution of ECM in the interstitial areas and on the surface of the scaffold, compared with PGS group. In contrast, many cavities and a heterogeneous distribution of ECM could be observed in the PGS group [Fig. 3(g)].

**FIG. 4.**
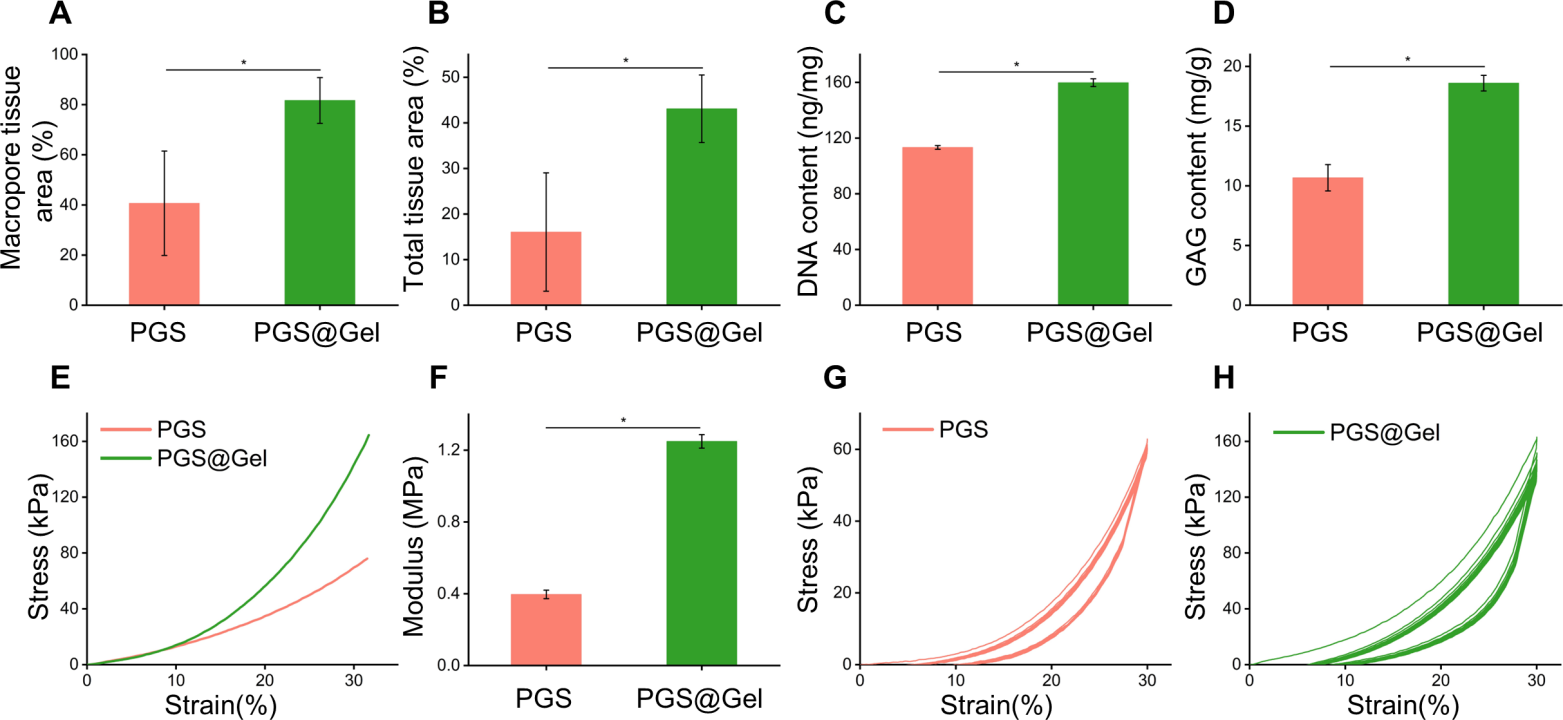
Quantitative analysis of the regenerated cartilage after 4 weeks *in vitro*: (a) tissue regeneration rate in macropores, (b) total tissue regeneration and integration rate, (c) DNA content, (d) GAG content, (e) stress–strain curves resulting from uniaxial compression tests, (f) comparison of compressive modulus, (g) and (h) cyclic compression tests of PGS, (g) and PGS@Gel (h) scaffolds. ^*^P < 0.05.

The quantitative results confirmed the image analysis observations. It was observed from HE staining that the tissue filling rate in the interstitial areas [PGS@Gel: 81.66 ± 9.12%, PGS: 40.67 ± 20.81%, Fig. 4(a)] as well as the total regenerated tissue rate of the PGS@Gel scaffolds (43.10 ± 7.41%) was significantly higher than that of the PGS scaffolds [16.06 ± 12.98%, Fig. 4(b)]. Furthermore, the biochemical ECM content (DNA content, GAG content) and mechanical properties (compression evaluation) of the regenerated cartilage formed with PGS@Gel scaffolds were significantly increased in comparison with the PGS scaffolds [Figs. 4(c)–4(h)]. Mechanical analysis indicated that the engineered constructs built by both the PGS and PGS@Gel scaffolds had good bioelasticity, fatigue durability, and improved stiffness. However, the PGS@Gel constructs had a higher elastic modulus (1.25 ± 0.04 MPa) than the PGS scaffold (0.40 ± 0.02 MPa), indicating that there was more ECM synthesis and deposition in the PGS@Gel scaffold [Fig. 4(f)].

Although the hydrophobicity and larger pore size of the PGS scaffold led to cell loss, some chondrocytes could still adhere and grow on the salt-leached rough surface of the filaments. By contrast, the PGS@Gel scaffold had more advantages than the PGS scaffold. First, chondrocyte adhesion, proliferation, migration, and ECM secretion were enhanced with the PGS@Gel scaffold, which can be attributed to the more appropriate pore size and improved hydrophilicity. More importantly, the macroscopic morphology of formed cartilage-like tissue was highly consistent with the 3D printed framework and was able to maintain its original shape. The favorable bioelasticity and fatigue durability of the cartilage-scaffold constructs can be attributed to the PGS bioelastomer; these properties are hardly provided by the types of polymeric biomaterials frequently processed by 3D printing (e.g., PLA, PGA, and PCL).

### C. *In vivo* cartilage regeneration

**FIG. 5.**
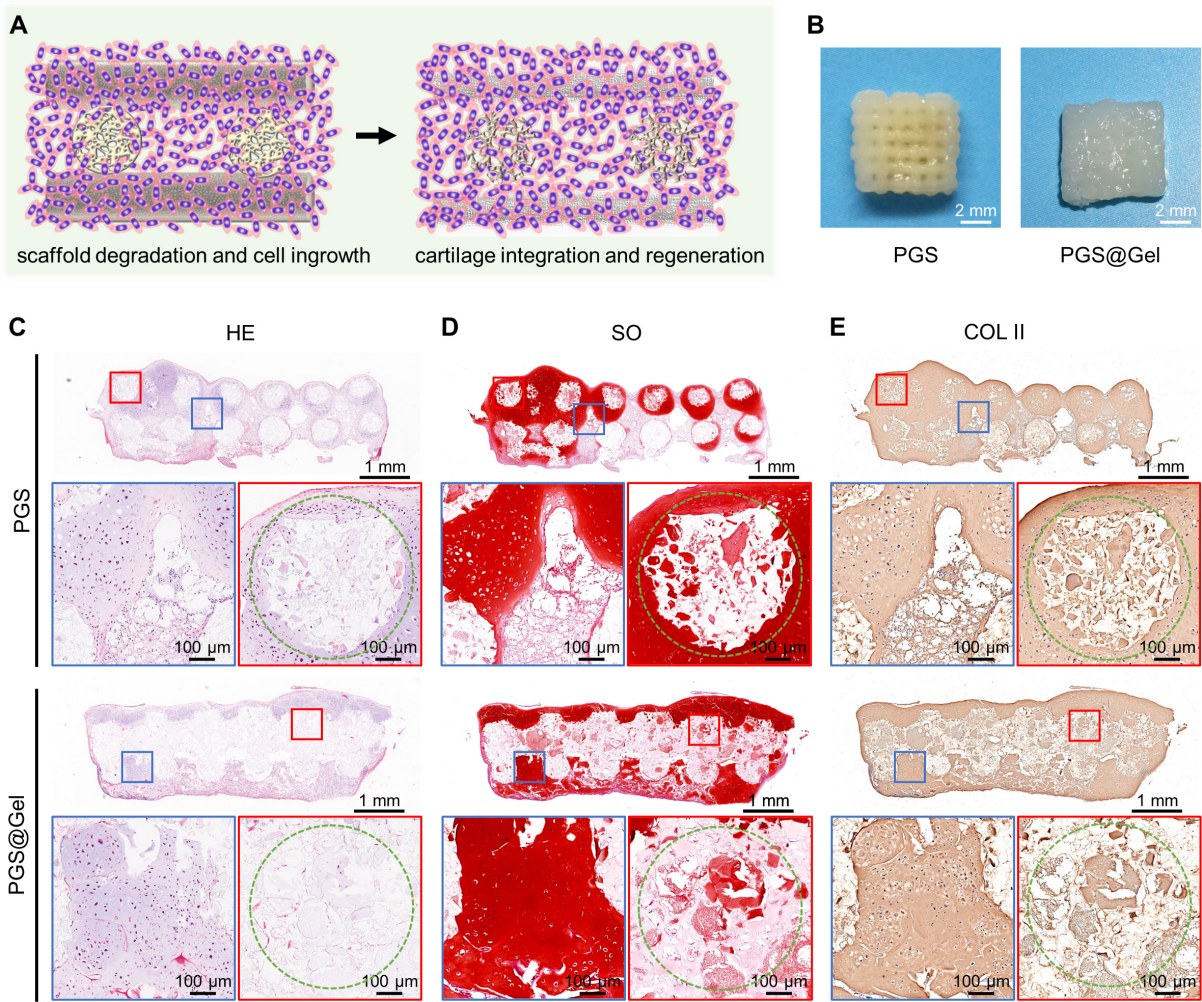
Gross observation and histological evaluations of regenerated cartilage 1 week after subcutaneous implantation in nude mice. (a) Schematic of cartilage formation accompanied by PGS framework degradation *in vivo*. (b) Optical images of engineered cartilages. Scale bar: 2 mm. (c)–(e) Hematoxylin and eosin (HE), Safranin-O (SO), and immunohistochemical collagen II (COL II) staining in sectional view. Blue rectangle: higher magnification of cartilage tissue in an interstitial area. Red rectangle: higher magnification of cartilage tissue in a filament area. Green circle: outline of scaffold filaments. Scale bar: 1 mm and 100 *μ*m.

To investigate the *in vivo* behavior of regenerated cartilage and its integration with scaffolds, following a 4-week culture period, the engineered cartilage was harvested and implanted subcutaneously in nude mice for 1 and 4 weeks. After 1-week subcutaneous implantation, cartilage-like tissue for both PGS and PGS@Gel groups gradually matured and kept their original shape well. The regenerated cartilage of the PGS@Gel scaffold was significantly denser than the PGS scaffold and had a similar ivory-white appearance of natural cartilage [Fig. 5(b)]. The sectional view of histological analysis demonstrated that the engineered cartilage of both groups was further mature with abundant cartilage-specific ECM as evidenced by HE, SO, and COL II [Figs. 5(c)–5(e)]. The PGS scaffold was partially degraded, and the PGS filament outline was obscure along with ingrowth of chondrocytes. Compared with the PGS group, the regenerated cartilage-like tissue of the PGS@Gel group was distributed evenly in the interstitial areas, was more mature with fusion tendency, and had better shape maintenance. Conversely, the distribution of the cartilage-like tissue in the PGS group was extremely uneven and a small amount was accompanied by some fibrous-like tissue within the interstitial areas.

**FIG. 6.**
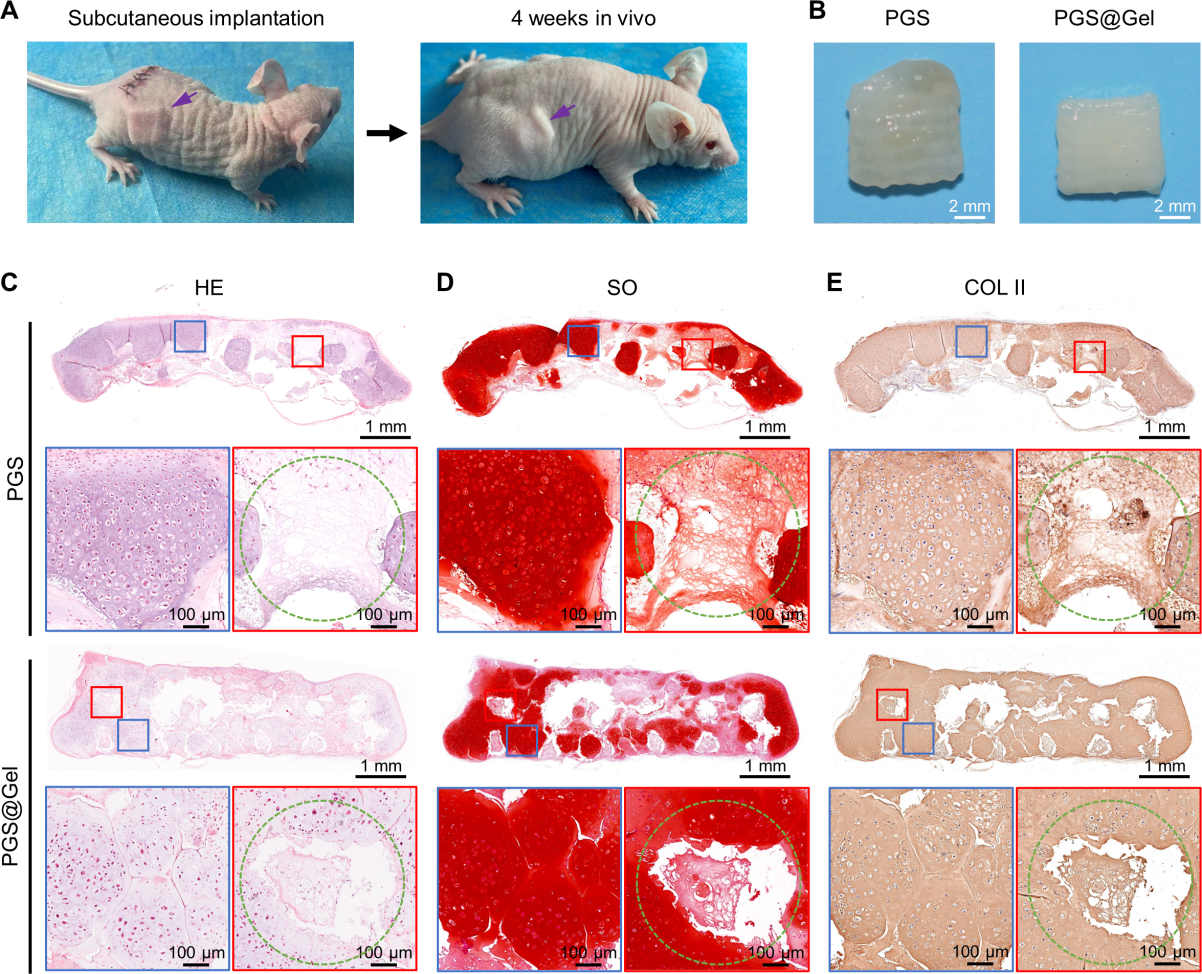
Histological evaluations of regenerated cartilage at 4 weeks after subcutaneous implantation in nude mice. (a) Regenerated cartilage under the skin of nude mice. (b) Optical images of engineered cartilages. Scale bar: 2 mm. (c)–(e) Hematoxylin and eosin (HE), Safranin-O (SO), and immunohistochemical collagen II (COL II) staining in sectional view. Blue rectangle: higher magnification of cartilage tissue in an interstitial area. Red rectangle: higher magnification of cartilage tissue in a filament area. Green circle: outline of scaffold filaments. Scale bar: 1 mm and 100 *μ*m.

After 4 weeks of dynamic adaptation *in vivo*, the regenerated cartilage-like tissue of both groups was more mature than after 1 week. The PGS@Gel group was still significantly superior to the PGS group, as it had a porcelain white appearance similar to natural cartilage tissue and its shape had not significantly changed [Figs. 6(a) and 6(b)]. A sectional view of the histological analysis revealed that the cartilage-like tissue formed in both groups was more mature and denser than after 1 week, and gradually integrated with the scaffold [Figs. 6(c)–6(e)]. It clearly showed that the PGS scaffold had been almost degraded and replaced by regenerated cartilage-like tissue and fibrous tissue. In the PGS@Gel group, newly formed cartilage-like tissue in the interstitial areas fused into one piece, and even the initial filament area had been replaced by cartilage-like tissue with little fibrous tissue. Additionally, the morphological structure of regenerated tissue was highly consistent with the original 3D printed PGS framework. However, the regenerated cartilage-like tissue of the PGS scaffold was only partially fused in the interstitial areas and mainly distributed on the surface of one side of the scaffold, accompanied by huge tissue cavities and fibrous tissue, as well as having poor consistency with the 3D printed framework.

The rough surface of the scaffolds was beneficial to the adhesion of chondrocytes, and chondrocytes migrated into PGS scaffolds along the interconnected micropores at an early stage. Interestingly, the cartilage regeneration rate aligned with the PGS degradation rate *in vivo*, as revealed by histological analysis. At 1-week post-implantation *in vivo*, the PGS scaffold was partially degraded, and indistinct filaments were invaded with a small number of chondrocytes [Fig. 5(c)]. Following 4 weeks of implantation, the regenerated cartilage in the interstitial areas was further matured and fused, meanwhile filaments were gradually replaced with cartilage and fibrous tissue [Fig. 6(c)].

**FIG. 7.**
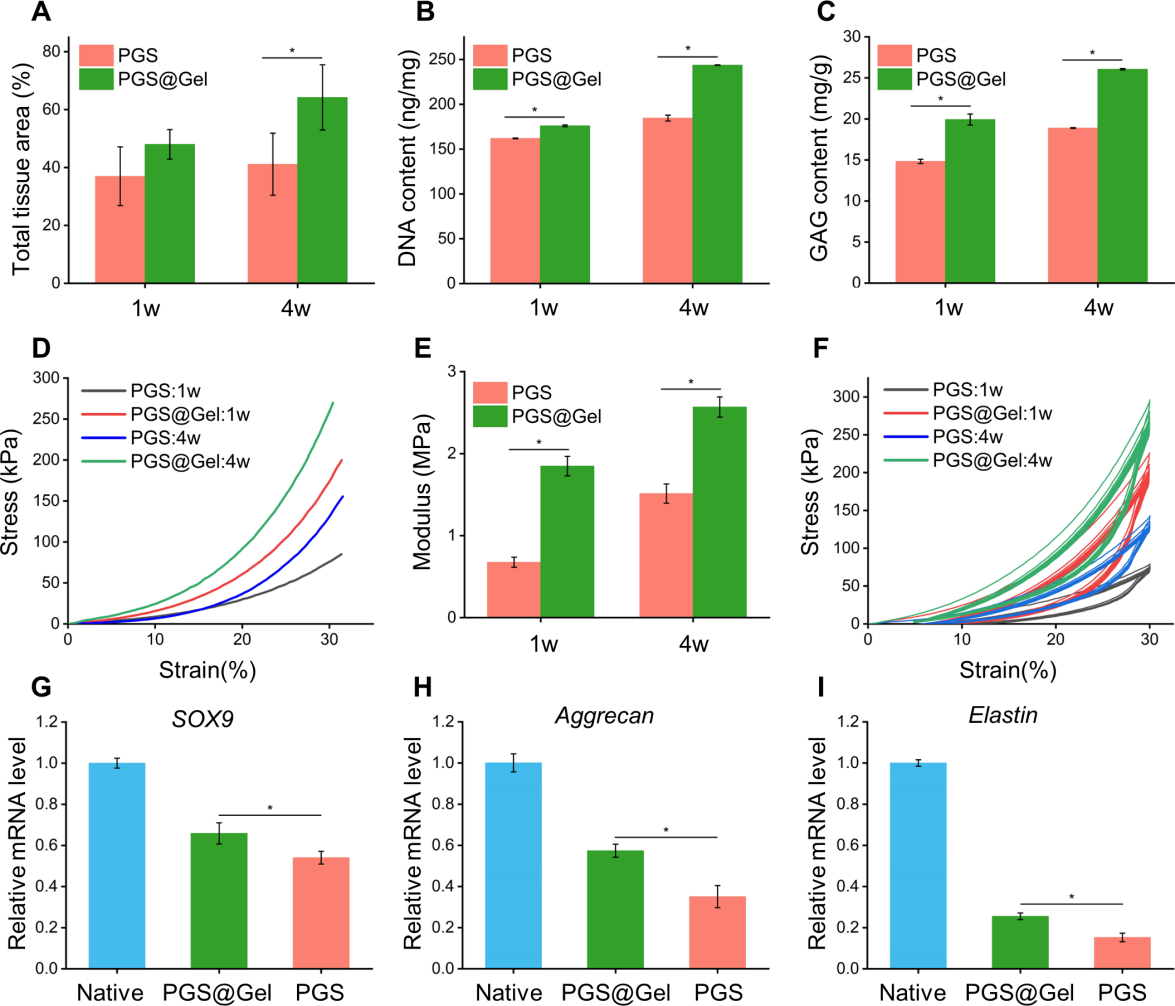
Quantitative and RT-qPCR analysis of the regenerated cartilage after 1- and 4-week subcutaneous implantation in nude mice: (a) total tissue regeneration and integration rate, (b) DNA content, (c) GAG content, (d) stress–strain curves resulting from uniaxial compression tests, (e) comparison of compressive modulus, (f) cyclic compression tests, and (g)–(i) RT-qPCR analysis of regenerated cartilage at 4 weeks *in vivo*. Determination of the expression of cartilage-related genes (SOX9 and aggrecan) and genes associated with elastic cartilage (elastin) via RT-qPCR using β-actin as the reference gene. The expression level of each gene in regenerated cartilage was compared with that of the native cartilage. ^*^P < 0.05.

Tissue regeneration and integration rate [Fig. 7(a)], biochemical ECM content (DNA content, GAG content), and mechanical properties (compression evaluations) all increased over the course of *in vivo* implantation. The DNA and GAG content analysis demonstrated that the quality of regenerated cartilage formed in the PGS@Gel group was superior to that of the PGS group [Figs. 7(b) and 7(c)]. Also, PGS@Gel had a higher elastic modulus than the PGS scaffold after *in vivo* implantation (PGS:0.68 ± 0.04, PGS@Gel:1.85 ± 0.12 MPa at 1 week *in vivo*), and the modulus increased with time [PGS:1.51 ± 0.11 MPa, PGS@Gel:2.59 ± 0.13 MPa at 4 weeks *in vivo*, Figs. 7(d)–7(f)]. Mechanical analysis revealed that the regenerated cartilage possessed outstanding bioelasticity and fatigue durability close to that of native cartilage and could sustain compressive stress cycles *in vivo*. Furthermore, RT-qPCR results confirmed that the expression levels of cartilage-specific genes expressed by PGS@Gel were higher than that of the PGS. The regenerated cartilages of PGS@Gel and PGS group both positively expressed specific elastin of elastic cartilage, which proved that the regenerated cartilages were elastic cartilage and still maintains their original phenotypes. Compared with the native elastic cartilage, the genes expression in regenerated cartilage was relatively low due to the shorter implanted time [Figs. 7(g)–7(i)]. It is believed that with the extension of the time *in vivo*, the elastin level will be close to that of the native.

Compared with the PGS@Gel scaffold, the cartilage regenerated with the PGS scaffold did not perform as well *in vitro* and *in vivo*; nevertheless, the PGS scaffold was able to support the regeneration of mature cartilage with elasticity. This can likely be attributed to the good biocompatibility of the PGS scaffold and the dynamic mechanical stimulus (having sustained compressive stress cycles under the skin of nude mice), which greatly facilitated cartilage regeneration *in vivo.*^37–40^ The regenerated cartilage of the PGS@Gel scaffold was mature and homogeneous with viscoelasticity similar to the native cartilage and had a high degree of integration with the 3D printed PGS framework. In addition to the dynamic mechanical stimulation, it also greatly benefited from the gelatin fibrous network. The gelatin network provided a more appropriate microporous structure and a modified hydrophilic surface to help promote chondrocyte adhesion and growth. Furthermore, the bioactivity of the PGS scaffolds was poor because there was only a small amount of hydroxyl groups in the molecular chain; however, the abundance of amino and carboxyl groups in the porous gelatin network improved the biological activity of the composite scaffolds to help guide chondrocyte fate.

Ideally, tissue integration should match the degradation rate of the biomaterial scaffold; however, some tissue cavities could still be observed in the PGS@Gel scaffold, which was caused by an imperfect synchronization of cartilage regeneration and the rapid degradation of the PGS (promoted by lipase). To address the relatively fast degradation rate of the PGS, adding PCL into the PGS/salt composite 3D printing ink could help control and increase the degradation rate of the PGS *in vivo*, as previously reported.^22,23^ As an essential component of biomaterial scaffolds, the delivery of bioactive molecules is a conventional and effective approach to promote tissue regeneration in the field of tissue engineering.^41^ However, limited by lack of active groups and the harsh cross-linking conditions (high temperature vacuum), drug loading of the 3D printed PGS scaffold is quite difficult. Here, gelatin-modified PGS@Gel scaffolds were produced by a classic freeze-drying method, and this combination may provide a convenient new approach for bioactive molecule delivery for 3D printed PGS scaffolds.^42^ Future research will focus on how to prepare functionalized elastic PGS scaffolds for efficiently specific tissue regeneration. Overall, these PGS@Gel scaffolds present a promising prospect for use in cartilage regeneration, with potential for adaptation for other tissue engineering applications.

## III. CONCLUSION

This study developed a composite PGS@Gel scaffold with gelatin fibrous networks and a built-in PGS framework, aiming to address the shortcomings of the standard 3D printed PGS scaffold. The PGS@Gel scaffold had a well-organized hierarchical porous structure, good hydrophilicity, outstanding elasticity, and fatigue durability, which helped obtain uniform, dense, mature, and shape-stable cartilage tissues with little scaffold residue. Although the regenerated cartilage had a simple blocky morphology, it is believed that artificial tissues such as auricular cartilage, nasal cartilage, and tracheal cartilage could be prepared through 3D printing of a bionic PGS@Gel scaffold. This method could also be easily applied to bioactive molecules or drugs to build specific functional microenvironments. In the future, we aim to design biomimetic scaffolds with cartilaginous bioactivity, or the ability to direct bone marrow mesenchymal stem cell differentiation, and investigate their potential for use in cartilage regeneration for clinical applications.

## IV. METHODS

### A. Preparation of the PGS/salt composite ink

In brief, PGS prepolymer was synthesized by polycondensation of an equimolar amount of glycerol (analytical grade, ≥99%, J&K Scientific Ltd.) and sebacic acid (analytical grade, ≥99%, J&K Scientific Ltd.) as previously described.^21^ Salt particles [sodium chloride (NaCl), 99.5%, Macklin] were sifted to get fine salts (≤76 *μ*m) after repeatedly grinding. Melted PGS prepolymer was dissolved in tetrahydrofuran (THF) (≥99.0%, Macklin) at a concentration of 50% (w/v). Sifted salt particles were added into the PGS prepolymer mixed solution at a 2:1 weight ratio (salt:PGS). Next, the mixture was placed into a drying oven at 70 °C for 4 h to remove most of the solvents. The obtained paste-like mixture was used as a composite ink in the following 3D printing procedure.

### B. Preparation of PGS and PGS@Gel scaffolds

#### 1. 3D printing of PGS scaffolds

The composited ink was continuously extruded and printed into PGS prepolymer/salt particle hybrid constructs with a multilayer structure by using a 3D bio-printer system (Regenovo3DBio-Architect). The nozzle's inner diameter was 500 *μ*m. Printing parameters of crisscrossed scaffold was as follows: the center-to-center distance between filaments was 1.2 mm with 0°/90° lay-down patterns between two successive layers, and the height of every layer was 450 *μ*m. The printed PGS prepolymer/salt particle constructs were then transferred to a vacuum oven and cured at 100 °C (0.5 bar) for 12 h and then further cured at 150 °C (1 bar) for 24 h. After curing, the resultant PGS scaffolds were soaked in distilled water for 12 h to remove the salt, with the water changed three times. Finally, the highly porous PGS scaffolds were freeze-dried in a vacuum.

#### 2. Fabrication of PGS@Gel scaffolds

To incorporate gelatin into the PGS scaffolds, 0.6% (w/v) type A gelatin (gel strength ∼240 g Bloom, Aladdin, Shanghai, China) was completely dissolved in de-ionized water. The 3D printed PGS scaffolds were immersed in the gelatin solution at 4 °C for 6 h, and then at −20 °C for 12 h. Next, after 48 h of lyophilization, the samples were crosslinked using the N-(3-dimethylaminopropyl)-N′-ethyl-carbodiimide hydrochloride (EDC) and N-hydroxysuccinimide (NHS) method for 24 h (EDC and NHS were dissolved in 98% ethanol, the concentrations of EDC and NHS were 0.5% and 0.3%, respectively, purchased from Aladdin). The residual solution was removed completely in de-ionized water. Finally, PGS@Gel scaffolds were obtained after being freeze-dried in a vacuum, then sterilized with ethylene oxide for future use.

### C. Characteristics of PGS and PGS@Gel scaffolds

#### 1. Structural characterization

A total view of the PGS and PGS@Gel scaffolds was recorded by camera and microscope. Then, the PGS and PGS@Gel scaffolds were sputtered with gold and subsequently observed in top view and sectional view at different magnifications using field emission scanning electron microscopy (FE-SEM, Hitachi, SU8010) with an accelerating voltage of 10 kV. The filament diameter and pore size were calculated according to the acquired SEM images using ImageJ.

#### 2. Porosity analysis

The porosity of PGS and PGS@Gel scaffolds was determined using the drainage method. Briefly, the initial weight of the dry scaffolds was recorded as W_d_ (n = 4 per group). Then, they were placed in absolute ethanol and the wet weight of scaffolds was recorded as W_w_. The porosity of each scaffold was calculated by the following equation: porosity = (W_w_ − W_d_)/(ρ × V) × 100%, where ρ and V represent the density of the ethanol and the volume of the scaffold, respectively.

#### 3. Swelling ratio evaluation

The swelling tests of scaffolds were evaluated by a volumetric ratio method, and the initial volume of the scaffold was referred to as V_0_. Subsequently, the dry scaffolds (n = 4 per group) were fully submerged in the PBS buffer solution, as taken out at the time points (2.0, 4.0, 8.0, 24.0, and 48.0 h). After swelling, the volume was referred to as Vs. The swelling ratio = Vs_/_V_0_.

#### 4. Mechanical properties

The mechanical properties of the PGS and PGS@Gel scaffolds and regenerated cartilages were analyzed using a mechanical testing machine (Instron-5542) as reported previously.^43^ The compression rate during testing was 10 mm/s. In the axial compression test, the scaffolds were compressed to maximal strain of 50%, and the regenerated cartilage samples were compressed to maximal strain of 30%. The moduli were calculated on the basis of the slope of the stress–strain curves for statistical analysis (n = 6). In the cyclic compression tests, the scaffold specimens were compressed to a maximal strain of 50% and recovered to a strain of 10% for 10 cycles, and the regenerated cartilage samples were compressed to a maximal strain of 30% and recovered to a strain of 5% for 10 cycles.

### D. Rabbit chondrocyte isolation and culture

Both nude mice and New Zealand white rabbits were purchased from Shanghai Jiagan Experimental Animal Raising Farm. Chondrocytes were isolated from the auricular cartilage of New Zealand white rabbits. In accordance with the method established previously,^44^ fibrous tissue and the perichondrium were removed from the harvested cartilage, and then the cartilage was fragmented into 1 mm^2^ pieces using scissors. Next, the cartilage pieces were immersed and washed in PBS containing 1% antibiotic. Finally, the ceramic-color pieces were digested with 0.15% collagenase NB4 (Worthington Biochemical Corp., Freehold, New Jersey, USA) for 8 h at 37 °C. The isolated chondrocytes were cultured in Dulbecco's modified Eagle's medium (DMEM) (Gibco BRL, Grand Island, NY, United States) containing 10% fetal bovine serum (Gibco BRL) and 1% antibiotic-antimycotic (Gibco BRL). Chondrocytes were passaged when they reached 90% confluence and passage 2 (P2) cells were harvested for use in subsequent experiments.

### E. Biocompatibility of PGS and PGS@Gel scaffolds

A 100 *μ*l chondrocyte suspension with the concentration of 80 × 10^6^ cells/ml was evenly seeded onto the PGS and PGS@Gel scaffolds. Phalloidin staining was preformed to observe the morphology of chondrocytes within PGS and PGS@Gel scaffolds after 3 and 7 days of culture *in vitro*. Briefly, the samples were washed by PBS twice and fixed in 4% paraformaldehyde (PFA) for 15 min. Next, they were washed using PBS thrice and immersed into dilute Actin-Tracker Red working solution for 45 min, following the manufacturer's instructions. The tracked samples were washed in PBS thrice. DAPI staining was carried out to detect nuclei. All images were observed and acquired by a Leica Thunder (Leica, DMi8).

### F. Tissue engineered cartilage regeneration *in vitro* and *in vivo*

Aforementioned cell-scaffold constructs were cultured in chondrogenic medium. The medium was changed every 2 days, and the tissue engineered cartilage was harvested after 4 weeks of cultivation *in vitro*. Subsequently, the engineered cartilage was implanted subcutaneously into nude mice (6 weeks old, female). Regenerated cartilage was obtained after 1 and 4 weeks post-implantation in nude mice.

### G. Real-time quantitative polymerase chain reaction (RT-qPCR) analysis

The expression of chondrogenesis-related genes (SOX9 and aggrecan) and elastic cartilage-related genes (elastin) was determined by RT-qPCR using β-actin as the reference gene for regenerated cartilage at 4 weeks *in vivo*. Briefly, total RNA was extracted from the regenerated cartilage of PGS, PGS@Gel, and native ear cartilage with TRIzol reagent (Invitrogen). The cDNA was obtained by reverse transcription (RT) using M-MLV 5 × reaction buffer (Promega), following the manufacturer's instructions. Subsequently, RT-qPCR was performed using the SYBR Premix Ex TaqTM II (Takara). Furthermore, the acquired results were analyzed using an Applied Biosystems AB instrument, and all tests were performed in triplicate. The primer sequences are listed in Table I.

**TABLE I.** Primer sequences of related genes.

Gene name	Primer sequences	Product length (bp)
*Aggrecan*	5′ CAC CCC GAG AAT CAA ATG GA 3′ 5′ TGG GCA GCG AGA CCT TGT 3′	116
*SOX9*	5′ CCA CCT CTC TTA CCT CTC TCA T 3′ 5′ AGG GTT TCT GTC TTG GAA GTT 3′	88
*Elastin*	5′ GCC TTT GGA GGT GTG TCT 3′ 5′ CAG CAG CAC CGT ATT TG 3′	58
*β-actin*	5′ GCA GAA ACG AGA CGA GAT TG 3′ 5′ GCA GAA CTT TGG GGA CTT TG 3′	167

### H. Histological and immunohistochemical analyses

Both *in vitro* and *in vivo* regenerated cartilage specimens were fixed in 4% paraformaldehyde, embedded in paraffin, and sectioned into 5-*μ*m sections. Hematoxylin and eosin (HE) and Safranin O (SO) staining was performed to observe the histological structure and glycosaminoglycan (GAG) deposition, as previously described.^45^ To analyze the expression of collagen II, a rabbit anti-human monoclonal antibody against collagen II (COL II) was used with a horseradish peroxidase (HRP)-conjugated anti-rabbit antibody (1:400 in PBS, Santa Cruz) as the secondary antibody. The HE-stained images were analyzed to evaluate the regenerated tissue percentage using ImageJ.

### I. Quantitative analysis

Quantitative analysis of the *in vitro* and *in vivo* regenerated cartilage was performed as described previously.^46^ GAG, DNA, and total collagen content in the samples (n = 3) were quantified using an Alcian Blue method, a Pico Green dsDNA assay (Invitrogen), and a hydroxyproline assay kit (Sigma Aldrich), respectively. The moduli were calculated by analyzing the stress–strain curves (n = 6).

### J. Statistical analysis

Data were analyzed using the GraphPad Prism 8 software. All values were reported as mean ± standard deviation. One-way analysis of variance was used to evaluate the statistically significant differences between groups. P values less than 0.05 were considered statistically significant.

## Data Availability

The data that support the findings of this study are available from the corresponding authors upon reasonable request.
